# Enabling Training of Neural Networks on Noisy Hardware

**DOI:** 10.3389/frai.2021.699148

**Published:** 2021-09-09

**Authors:** Tayfun Gokmen

**Affiliations:** IBM Research AI, Yorktown Heights, NY, United States

**Keywords:** learning algorithms, training algorithms, neural network acceleration, Bayesian neural network, in-memory computing, on-chip learning, crossbar arrays, memristor

## Abstract

Deep neural networks (DNNs) are typically trained using the conventional stochastic gradient descent (SGD) algorithm. However, SGD performs poorly when applied to train networks on non-ideal analog hardware composed of resistive device arrays with non-symmetric conductance modulation characteristics. Recently we proposed a new algorithm, the Tiki-Taka algorithm, that overcomes this stringent symmetry requirement. Here we build on top of Tiki-Taka and describe a more robust algorithm that further relaxes other stringent hardware requirements. This more robust second version of the Tiki-Taka algorithm (referred to as TTv2) 1. decreases the number of device conductance states requirement from 1000s of states to only 10s of states, 2. increases the noise tolerance to the device conductance modulations by about 100x, and 3. increases the noise tolerance to the matrix-vector multiplication performed by the analog arrays by about 10x. Empirical simulation results show that TTv2 can train various neural networks close to their ideal accuracy even at extremely noisy hardware settings. TTv2 achieves these capabilities by complementing the original Tiki-Taka algorithm with lightweight and low computational complexity digital filtering operations performed outside the analog arrays. Therefore, the implementation cost of TTv2 compared to SGD and Tiki-Taka is minimal, and it maintains the usual power and speed benefits of using analog hardware for training workloads. Here we also show how to extract the neural network from the analog hardware once the training is complete for further model deployment. Similar to Bayesian model averaging, we form analog hardware compatible averages over the neural network weights derived from TTv2 iterates. This model average then can be transferred to another analog or digital hardware with notable improvements in test accuracy, transcending the trained model itself. In short, we describe an end-to-end training and model extraction technique for extremely noisy crossbar-based analog hardware that can be used to accelerate DNN training workloads and match the performance of full-precision SGD.

## Introduction

Deep neural networks (DNNs) (LeCun et al., 2015) have achieved tremendous success in multiple domains outperforming other approaches and even humans (He et al., 2015) at many problems: object recognition, video analysis, and natural language processing are only a few to mention. However, this success was enabled mainly by scaling the DNNs and datasets to extreme sizes, and therefore, it came at the expense of needing immense computation power and time. For instance, the amount of compute required to train a single GPT-3 model composed of 175B parameters is tremendous: 3,600 Petaflops/s-days (Brown et al., 2020), equivalent to running 1,000 state-of-the-art NVIDIA A100 GPUs, each delivering 150 Teraflops/s performance for about 24 days. Hence, today’s and tomorrow’s large models are costly to train both financially and environmentally on currently available hardware (Strubell et al., 2019), begging for faster and more energy-efficient solutions.

DNNs are typically trained using the conventional stochastic gradient descent (SGD) and backpropagation (BP) algorithm (Rumelhart et al., 1986). During DNN training, matrix-matrix multiplications; hence repeated multiply and add operations dominate the total workload. Therefore, regardless of the underlying technology, realizing highly optimized multiply and add units and sustaining many of these units with appropriate data paths is practically the only game everybody plays while proposing new hardware for DNN training workloads (Sze et al., 2017).

One approach that has been quite successful in the past few years is to design highly optimized digital circuits using the conventional CMOS technology that leverages reduced-precision arithmetic for the multiply and add operations. These techniques are already employed to some extent by current GPUs (Nvidia, 2021) and other application-specific-integrated-circuits (ASIC) designs, such as TPUs (Cloud TPU, 2007) and IPUs (Graphcore, 2021). There are also many research efforts extending the boundaries of the reduced precision training, using hybrid 8-bit (Sun et al., 2019) and 4-bit (Sun et al., 2020) floating-point and 5-bit logarithmically scaled (Miyashita et al., 2016) number formats.

Alternative to digital CMOS, hardware architectures composed of novel resistive cross-point device arrays have been proposed that can deliver significant power and speed benefits for DNN training (Gokmen and Vlasov, 2016; Haensch et al., 2019; Burr et al., 2017; Burr et al., 2015; Yu, 2018). We refer to these cross-point devices as resistive processing unit [RPU (Gokmen and Vlasov, 2016)] devices as they can perform all the multiply and add operations needed for training by relying on physics. Out of all multiply and add operations during training, 1/3 are performed during forward propagation, 1/3 are performed during error backpropagation, and finally, 1/3 are performed during gradient computation. RPU devices use Ohm’s law and Kirchhoff’s law (Steinbuch, 1961) to perform the multiply and add needed for the forward propagation and error backpropagation. However, more importantly, *RPUs use the device conductance modulation and memory characteristics to perform the multiply and add needed during the gradient computation* (Gokmen and Vlasov, 2016)*.*

Unfortunately, RPU based crossbar architectures have had only minimal success so far. That is mainly because the training accuracy on this imminent analog hardware strongly depends on the cross-point elements’ conductance modulation characteristics when the conventional SGD algorithm is used. One of the key requirements is that these devices must symmetrically change conductance when subjected to positive or negative pulse stimuli (Gokmen and Vlasov, 2016; Agarwal et al., 2016). Theoretically, it is shown that only symmetric devices provide an unbiased gradient calculation and accumulation needed for the SGD algorithm. Whereas any non-symmetric device characteristic modifies the optimization objective and hampers the convergence of SGD based training (Gokmen and Haensch, 2020; Onen et al., 2021).

Many different solutions are proposed to tackle the SGD’s converge problem on crossbar arrays. First, widespread efforts to engineer resistive devices with symmetric modulation characteristics have been made (Fuller et al., 2019; Woo and Yu, 2018; Grollier et al., 2020), but a mature device technology with the desired behavior remains to be seen. Second, many high-level mitigation techniques have been proposed to overcome the device asymmetry problem. One critical issue with these techniques is the serial access to cross-point elements either one-by-one or row-by-row (Ambrogio et al., 2018; Agarwal et al., 2017; Yu et al., 2015). Serial operations such as reading conductance values individually, engineering update pulses to force symmetric modulation artificially, and carrying or resetting weights periodically come with a tremendous overhead for large networks. Alternatively, there are approaches that perform the gradient computation outside the arrays using digital processing (Nandakumar et al., 2020). Note that irrespective of the DNN architecture, 1/3 of the whole training workload is in the gradient computation. For instance, for the GPT-3 network, 1,200 Petaflops/s-days are required solely for gradient computation throughout the training. Consequently, these approaches cannot deliver much more performance than the fully digital reduced-precision alternatives mentioned above. In short, there exist solutions possibly addressing the convergence issue of SGD on non-symmetric device arrays. However, they all defeat the purpose of performing the multiply and add operations on the RPU device and lose the performance benefits.

In contrast to all previous approaches, we recently proposed a new training algorithm, the so-called Tiki-Taka algorithm (Gokmen and Haensch, 2020), that performs all three cycles (forward propagation, error backpropagation, and gradient computation) on the RPU arrays using the physics and converges with non-symmetric device arrays. Tiki-Taka works very differently from SGD, and we showed in another study that non-symmetric device behavior plays a useful role in the convergence of Tiki-Taka (Onen et al., 2021).

Here, we build on top of Tiki-Taka and present a more robust second version that relaxes other stringent hardware issues by orders of magnitude, namely the limited number of states of RPU devices and noise. We refer to this more robust second version of the Tiki-Taka algorithm as TTv2 for the rest of the paper. In the first part of the paper, we focus on training and present TTv2 algorithm details and provide simulation results at various hardware settings. We tested TTv2 on various network architectures, including fully connected, convolutional, and LSTMs, although the presented results focus on the more challenging LSTM network. TTv2 shows significant improvements in the training accuracy compared to Tiki-Taka, even at much more challenging hardware settings. In the second part of the paper, we show an analog-hardware-friendly technique to extract the trained model from the noisy hardware. We also generalize this technique and apply it over TTv2 iterates and extract each weight’s time average from a particular training period. These weight averages provide a model that approximates the Bayesian model average, and it outperforms the trained model itself. *With this new training algorithm and accurate model extraction technique, we show that the noisy analog hardware composed of RPU device arrays can provide scalable training solutions that match the performance of full-precision SGD.*

### PART I: Training

In this section, we first give an overview of the device arrays and device update characteristics used for training. Then we present a brief background on Tiki-Taka. Finally, we detail TTv2 and provide comprehensive simulation results on an LSTM network at various hardware settings.

### Device Arrays and Conductance Modulation Characteristics

Resistive crossbar array of devices performs efficient matrix-vector multiply (y=Wx) using Ohm’s law and Kirchhoff’s law. The device array’s stored conductance values form a matrix (W), whereas the input vector (x) is transmitted as voltage pulses through the columns, and the resulting vector (y) is read as current signals from the rows. However, only positive conductance values are allowed physically. Therefore, to encode both positive and negative matrix elements, a pair of devices is operated in differential mode. With the help of the peripheral circuits supplying the voltage inputs and reading out the differential current signals, logical matrix elements ($w_{ij}$) are mapped to physical conductance pairs as

$$w_{ij}=\text{Κ}\left(g_{ij}-g_{ij,ref}\right)$$ (1)

where Κ is a global gain factor controlled by the periphery, and $g_{ij}$ and $g_{ij,ref}$ are the conductance values stored at each pair corresponding to the *i*th row and *j*th column. Moreover, crossbar arrays can be easily operated in the transpose mode by changing the periphery’s input and output directions. As a result, a pair of arrays with the supporting peripheral circuits provide a logical matrix (also referred to as a single tile) that any algorithm can utilize to perform a series of matrix-vector multiplications (mat-vec) using W and $W^{T}$.

For training algorithms, the efficient update of the stored matrix elements is also an essential component. Therefore, device conductance modulation and memory characteristics are utilized to implement a local and parallel update on RPU arrays. During the update cycle, input signals are encoded as a series of voltage pulses and simultaneously supplied to the array’s rows and columns. Note that the voltage pulses are applied only to the first set of RPU devices, and the reference devices are kept constant. As a result of voltage pulse coincidence, the corresponding RPU device changes its conductance by a small amount bi-directionally, depending on the voltage polarity. This incremental change in device conductance results in an incremental change in the stored weight value, and the RPU response is governed by Eq. 2.

$$w_{ij}\leftarrow w_{ij}\mp \text{Δ}w_{min,ij}F_{ij}\left(w_{ij}\right)-\left|\text{Δ}w_{min,ij}\right|G_{ij}\left(w_{ij}\right)$$ (2)

In Eq. 2, ∓ sign is decided by the external voltage pulse polarity, whereas $\text{Δ}w_{min,ij}$ is the incremental weight change due to single pulse coincidence, and $F_{ij}\left(w_{ij}\right)$ and $G_{ij}\left(w_{ij}\right)$ are the symmetric (additive) and antisymmetric (subtractive) combinations of the positive and negative conductance modulation characteristics (Gokmen and Haensch, 2020), all of which are the properties of the updated device corresponding to the *i*th row and *j*th column. Eq. 2 is very general and governs the computation (hardware-induced update) performed by the tile for all sorts of RPU device behaviors, assuming the device conductance modulation characteristics are some function of the device conductance state. If the conductance modulations are much smaller than the whole conductance range of operation, Eq. 3 can be derived from Eq. 2.

$$w_{ij}\leftarrow w_{ij}+\eta \left[\delta_{i}\times x_{j}\right]F_{ij}\left(w_{ij}\right)-\eta \left|\left[\delta_{i}\times x_{j}\right]\right|G_{ij}\left(w_{ij}\right)$$ (3)

In Eq. 3, $x_{j}$ and $\delta_{i}$ represent the input values used for updates for each column and row, respectively corresponding to activations and errors calculated in the forward and backward cycles, and η is a scalar controlling the strength of the update, all of which are inputs to pulse generation circuitry at the periphery. Here, we use the stochastic pulsing scheme proposed in Ref Gokmen and Vlasov (2016), and during the parallel update, the number of pulses generated by the periphery is bounded by $n_{pulse}=\lceil \eta \text{max}\left(\left|\delta_{i}\right|\right)\text{max}\left(|x_{j}|\right)/\mu_{\text{Δ}w}\rceil$, where $\mu_{\text{Δ}w}$ is the mean of $\text{Δ}w_{min,ij}$ for the whole tile. Using $n_{pulse}$ stochastic translators generate pulses with the correct probability; therefore, Eq. 3 is valid in expectation. Whereas in the limit of a single pulse coincidence, the RPU response is governed by Eq. 2.

**FIGURE 1:**
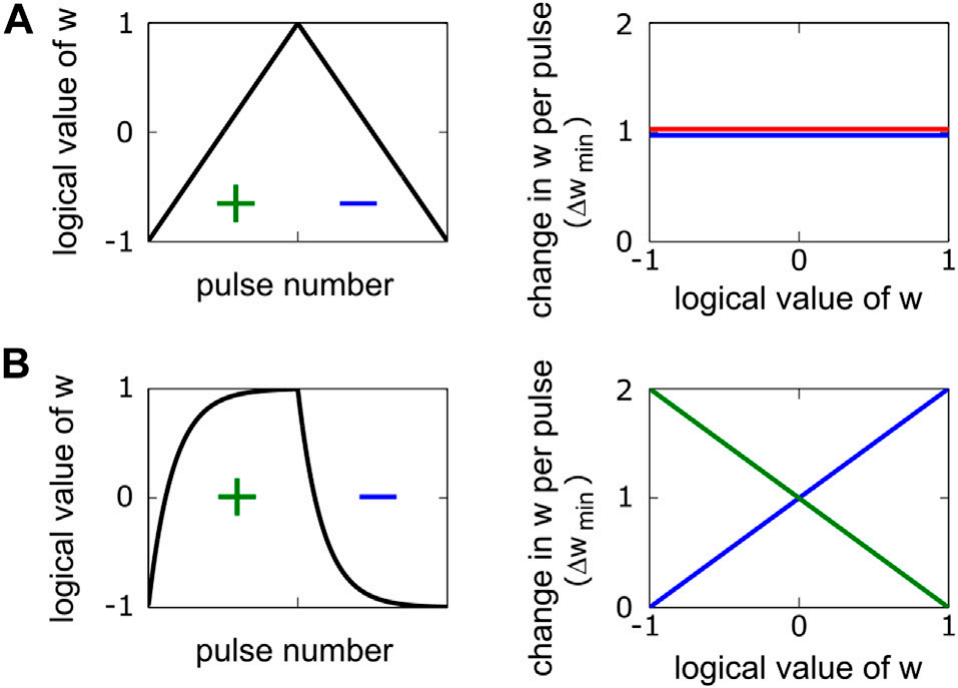
Pulse responses and weight modulation characteristics are illustrated for two different devices. **(A)** Symmetric and linear device: Weight increments (red) and decrements (blue) are equal in size and do not depend on the weight. **(B)** Exponentially saturating device: Weight increments and decrements both have linear dependencies on the weight. However, there exists a single weight value at which the strengths of the weight increment and decrement are equal. This point is called the symmetry point, and it is at w=0 for the illustrated example.

Figure 1A illustrates a pulse response of a linear and symmetric device, where F(w)=1 and G(w)=0, and the hardware-induced update rule simplifies to the SGD update rule of $w_{ij}\leftarrow w_{ij}+\eta \left[\delta_{i}\times x_{j}\right]$. In the literature, this kind of device behavior is usually referred to as the “ideal” device required for SGD. For a non-linear but symmetric device, F(w) deviates from unity and becomes a function of w, but G(w) remains zero. For non-symmetric devices, G(w) also deviates from zero and becomes a function of w, hence differing from the form required by SGD. Figure 1B illustrates an exponentially saturating non-symmetric device where $w_{ij}\leftarrow w_{ij}+\eta \left[\delta_{i}\times x_{j}\right]-\eta \left|\left[\delta_{i}\times x_{j}\right]\right|w$ provides the computation performed by this device. Although this form of update behavior causes convergence issues for SGD, Tiki-Taka trains DNNs successfully with all sorts of non-symmetric devices (Gokmen and Haensch, 2020). Therefore, in contrast to SGD, all sorts of non-symmetric device behaviors can be considered “ideal” for Tiki-Taka.

Tiki-Taka’s training performance depends on the successful application of the symmetry point shifting technique (Kim et al., 2019), which guarantees G(w=0)=0 for all elements in the tile. This behavior is illustrated for the device in Figure 1B, where the strengths of the positive and negative weight increments are equal in size at w=0. The symmetry point shifting is achieved by programming the reference device conductance to a value corresponding to the updated device’s symmetry point. For the rest of the paper, we assume the symmetry point shifting is also applied in the context of TTv2. Although we developed techniques to eliminate this requirement, it is beyond the scope of this paper and will be published elsewhere.

### Algorithms

**FIGURE 2:**
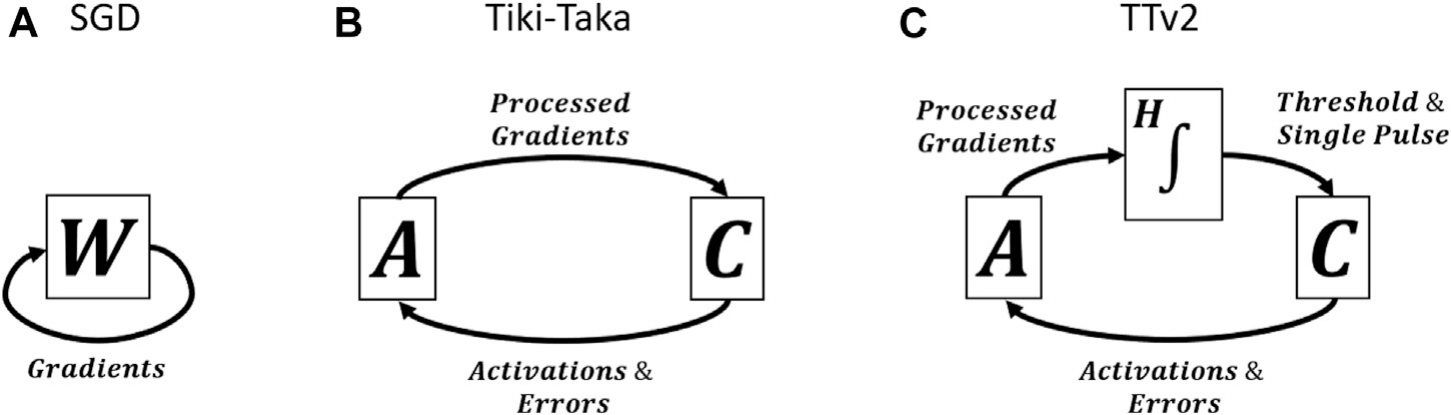
Schematics of SGD, Tiki-Taka, and TTv2 dynamics.

SGD, Tiki-Taka, and TTv2 all use the error backpropagation, but they process the gradient information differently and hence are fundamentally distinct algorithms. Figures 2A,B show schematics of SGD and Tiki-Taka dynamics (iterations), respectively. Tiki-Taka replaces each weight matrix W of SGD with two matrices (referred to as matrix A and C) and creates a coupled dynamical system by exchanging information between the two. As shown in Ref Onen et al. (2021), the non-symmetric behavior is a valuable and required property of the device in the Tiki-Taka dynamics. During the information exchange between the two systems, device asymmetry creates a dissipation mechanism, resulting in minimization of the system’s total energy (Hamiltonian); hence Tiki-Taka is also called Stochastic Hamiltonian Descent (Onen et al., 2021). However, the noise introduced during the transfer of the information (processed gradients) from A to C caused additional test error for Tiki-Taka and needed to be addressed (Gokmen and Haensch, 2020).

The schematic in Figure 2C illustrates the TTv2 dynamics, highlighting our main contribution. TTv2 introduces an additional stage (H), between the transfer from A to C, which performs integration in the digital domain, providing a low pass filtering function. Furthermore, the model’s parameters are stored solely on C and only updated if H reaches a threshold value. Because of these modifications in TTv2, the model’s parameters are updated more slowly but with higher confidence, bringing significant benefits against various hardware noise issues. Details of the algorithm are provided below.

### Tiki-Taka Algorithm

**Algorithm 1** outlines the details of the Tiki-Taka algorithm. Tiki-Taka uses two matrices, A and C, and the neural network parameters are defined by W=γA+C, where γ is a scalar hyperparameter set between [0,1]. Using W, Tiki-Taka computes the activations (x) and the error signals (δ) by utilizing the conventional backpropagation algorithm. The activation and error computations are identical to SGD and therefore omitted from the algorithm description. Also, there are multiple layers, but **Algorithm 1** only illustrates the operations performed on a single layer for simplicity. After performing the forward propagation and the error backpropagation on A and C (lines 8 and 9), Tiki-Taka updates only A by employing the hardware-induced parallel update (line 10) using x and δ. $\eta_{a}$ is the learning rate used for updating A. These operations are repeated for ns times, a hyperparameter of Tiki-Taka. After every ns update on A, an analog mat-vec is performed on A with an input vector u, resulting in a vector v (line 14). The vector u is generated each time locally, and it is either a one-hot encoded vector or a column vector of a Hadamard matrix used in a cyclic fashion. Using the generated u vector and the result of f(v), C is updated by employing the hardware-induced parallel update (line 15). f(v) is a pointwise function: $f\left(v_{i}\right)=\{\begin{array}{cc} v_{i}, & \text{if}\;\left|v_{i}\right|\geq T\\ 0, & \text{otherwise} \end{array}$ where T is set to the mat-vec noise. $\eta_{c}$ is the learning rate used for updating C. These operations are repeated for the data examples in the training dataset for multiple epochs until a converge criteria is met. Following the same practices described in Ref Gokmen and Haensch (2020), here we also use the one-hot encoded u vectors and the thresholding f(v) for the LSTM simulations.


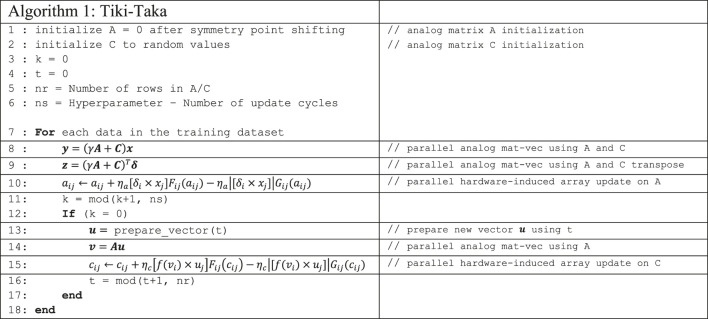


### TTv2 Algorithm

**Algorithm 2** outlines the details of the TTv2 algorithm. In addition to A and C matrices allocated on analog arrays, TTv2 also allocates another matrix H in the digital domain. This matrix H is used to implement a low pass filter while transferring the gradient information processed by A to C. In contrast to Tiki-Taka, TTv2 uses only the C matrix to encode the neural network’s parameters, corresponding to γ=0. Therefore, the activation (x) and error (δ) computations are performed using C (lines 10 and 11). TTv2 does not change the updates performed on A. After ns updates, a mat-vec is performed on A. Unlike Tiki-Taka, TTv2 only uses a one-hot encoded u vector while performing a mat-vec on A. This provides a noisy estimate of a single row of A, and the results are stored in v. After this step, the significant distinction between Tiki-Taka and TTv2 appears. Instead of using u and v to update C, TTv2 first accumulates v (after scaling with $\eta_{c}$) on H’s corresponding row, referred to as H(row = t). During this digital vector-vector addition, the magnitude of any element in H(row = t) may exceed unity. In that case, the corresponding elements are reset back to zero, and a single pulse parallel update on C is performed. The C update of TTv2 uses the sign information of the elements that grew in amplitude beyond one and the row information t. After these steps, TTv2 loops back and repeats these operations for other data examples until it reaches convergence.


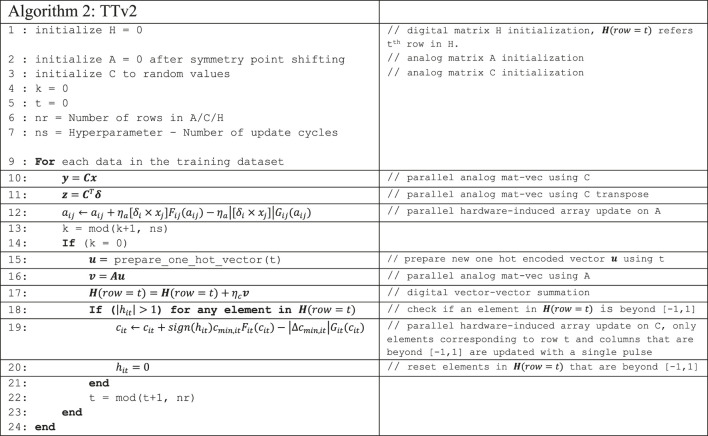


### Array Model

We use a device model like the one presented in Figure 1B but with significant array level variability and noise for the training simulations. We simulate stochastic translators at the periphery during the update, and each coincidence event triggers an incremental weight change on the corresponding RPU as described below. We also introduce noise and signal bounds during the matrix-vector multiplications performed on the arrays.

During the update, the weight increments ($\Delta w_{ij}^{+}$) and decrements ($\Delta w_{ij}^{-}$) are assumed to be functions of the current weight value. For the positive branch $\Delta w_{ij}^{+}=\Delta w_{min,ij}\left(1-slope_{ij}^{+}\times w_{ij}\right)$ and for the negative branch $\Delta w_{ij}^{-}=\Delta w_{min,ij}\left(1+slope_{ij}^{-}\times w_{ij}\right)$, where $slope_{ij}^{+}$ and $slope_{ij}^{-}$ are the slopes that control the dependence of the weight changes on the current weight values, and $\Delta w_{min,ij}$ is the weight change due to a single coincidence event at the symmetry point. This model results in three unique parameters for each RPU element. All these parameters are sampled independently using a unit Gaussian random variable and then used throughout the training, providing device-to-device variability. The slopes are obtained using $slope_{ij}^{+}=\mu_{s}\left(1+\sigma_{s}\xi_{ij}^{+}\right)$ and $slope_{ij}^{-}=\mu_{s}\left(1+\sigma_{s}\xi_{ij}^{-}\right)$, where $\mu_{s}=1.66$, $\sigma_{s}$ is set to 0.1, 0.2, or 0.3 for different experiments, and ξ are the independent random samples. The simulation results were insensitive to $\sigma_{s}$; therefore, we only show results corresponding to $\sigma_{s}=0.2$. The weight increments at the symmetry point are obtained using $\Delta w_{min,ij}=\mu_{\Delta w}\left(1+\sigma_{\Delta w}\xi_{ij}\right)$, where $\sigma_{\Delta w}=0.3$ and $\mu_{\Delta w}$ is the array average varied from 0.6 × 10^−4^ up to 0.15 for different experiments to study the effects number of states on training accuracy. We define the number of states as the ratio of the nominal weight range to the nominal weight increment at the symmetry point; therefore, $2/\left(\mu_{s}\mu_{\Delta w}\right)$ provides the average number of states. Note that this definition of the number of states is very different from the definition used for devices developed for memory applications, and it should not be compared against multi-bit storage elements. Besides, additional Gaussian noise is introduced to each weight increment and decrement to capture the cycle-to-cycle noise: For the multiplicative noise model $\Delta w_{ij}^{\mp }\to \Delta w_{ij}^{\mp }\left(1+\sigma_{cycle}\xi \right)$, whereas for the additive noise model $\Delta w_{ij}^{\mp }\to \Delta w_{ij}^{\mp }+\Delta w_{min,ij}\sigma_{cycle}\xi$, where $\sigma_{cycle}$ is set to 0.3 or 1 for different experiments, and ξ is sampled from a unit Gaussian for each coincidence event.

During the matrix-vector multiplications, we inject additive Gaussian noise into each output line to account for analog noise. Therefore, the model becomes $y=\mathrm{Wx}+\sigma_{MV}\xi$, where $\sigma_{MV}=0.06$, corresponding to 10% of the nominal weight maximum ($1/\mu_{s}$). Moreover, the matrix-vector multiplications are bounded to 20 times the nominal weight maximum to account for signal saturation at the output lines. The input signals are assumed to be between [−1, 1] with a 7-bit input resolution, whereas the outputs are quantized assuming a 9-bit ADC. To mitigate the shortcomings of the signal bounds, we use the noise, bound, and update management techniques described in Ref Gokmen et al. (2017).

### Training Simulations

We performed training simulations for fully connected, convolutional, and LSTM networks: the same three networks and datasets studied in Ref Gokmen and Haensch (2020). However, the presented results focus on the most challenging LSTM network referred to as LSTM2-64-WP in Ref Gokmen et al. (2018). This network is composed of two stacked LSTM blocks, each with a hidden state number of 64. Leo Tolstoy’s War and Peace (WP) novel is used as a dataset, and it is split into training and test sets as 2,933,246 and 325,000 characters with a total vocabulary of 87 characters. This task performs a character-based language model where the input to the network is a sequence of characters from the WP novel, and the network is trained with the cross-entropy loss function to predict the next character in the sequence. LSTM2-64-WP has three different weight matrices for SGD, and including the biases, they have sizes 256 × (64 + 87 + 1) and 256 × (64 + 64 + 1) for the two LSTM blocks and 87 × (64 + 1) for the fully connected layer before the softmax activation. Each matrix of SGD maps to two seperate A and C matrices for Tiki-Taka and TTv2.

**FIGURE 3:**
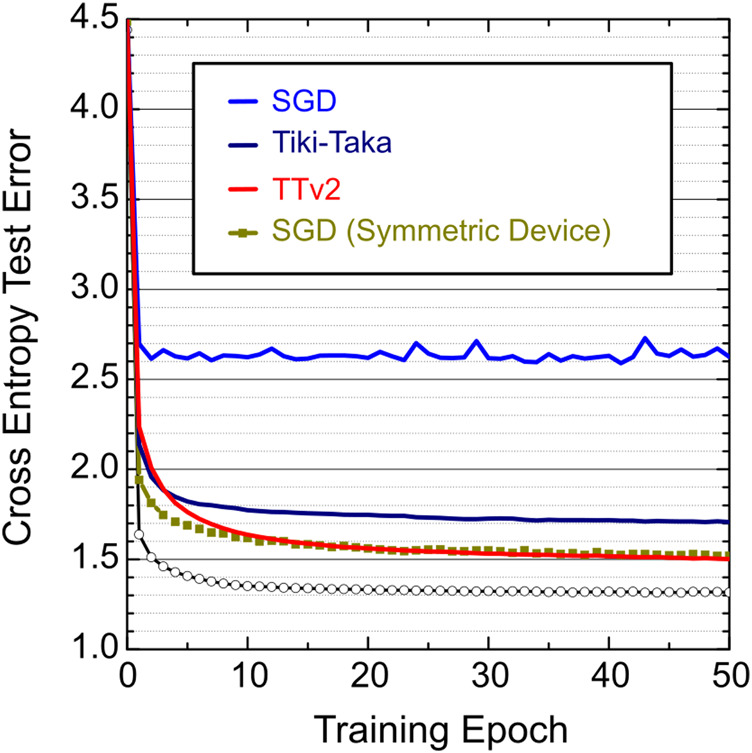
LSTM training simulations for SGD, Tiki-Taka, and TTv2 algorithms. Different color curves use an array model with non-symmetric devices, $\mu_{\Delta w}=0.001$ (corresponding to 1,200 states), and the multiplicative cycle-to-cycle update noise at $\sigma_{cycle}=0.3$. The square symbols show the SGD training using linear and symmetric devices where all devices’ slope parameters are set to zero while all other array parameters remain unchanged. The open circles are the floating-point baseline.

Figure 3 shows simulation results for SGD, Tiki-Taka, and TTv2 for non-symmetric device arrays with $\mu_{\Delta w}=0.001$ (corresponding to 1,200 average number of states) and the multiplicative cycle-to-cycle noise $\sigma_{cycle}=0.3$. Additionally, we simulate the SGD training using symmetric device arrays where all devices’ slope parameters are set to zero while all other array parameters remain unchanged. We also note that without changing the analog hardware settings, we virtually remap the nominal weight range from [−0.6, 0.6] to [−2, 2] using the digital scaling trick shown in Ref Rasch et al. (2020) for all LSTM simulations. This remapping slightly increases SGD and Tiki-Taka’s training performance compared to the results published in Ref Gokmen and Haensch (2020). We also optimized Tiki-Taka’s hyper-parameters to achieve the best possible training performance at this modified weight range.

In Figure 3, Tiki-Taka performs significantly better than SGD for non-symmetric devices, but a clear gap exists between the symmetric device SGD and the Tiki-Taka results. This gap is due to the noise during the analog mat-vec performed on A (line 14 of Tiki-Taka). Ref Gokmen and Haensch (2020) showed that the remaining gap closes if the noise during the mat-vec on A is reduced by 10x to $\sigma_{MV}=0.006$; however, this low noise setting is unrealistic for analog hardware. In contrast, TTv2 shows indistinguishable results compared to the symmetric device SGD, even when the mat-vec noise on A is at $\sigma_{MV}=0.06$. Therefore, these simulation results prove the benefits of introducing the filtering stage while transferring information from A to C, and TTv2 increases the algorithm’s noise tolerance to the mat-vec performed by the analog arrays at least by 10x compared to Tiki-Taka.

**FIGURE 4:**
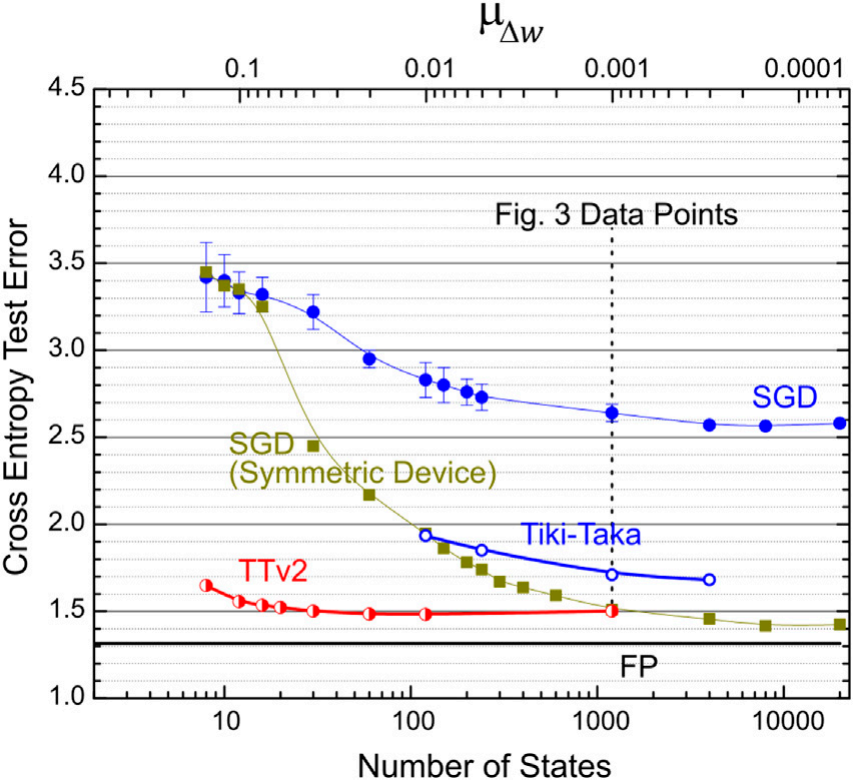
LSTM training simulations for SGD, Tiki-Taka, and TTv2 algorithms as a function of $\mu_{\Delta w}$. 10x increase in $\mu_{\Delta w}$ results in a simultaneous 10x reduction in the number of states and a 10x increase in the cycle-to-cycle update noise. Circles correspond to an array model with non-symmetric devices, whereas squares are for symmetric and linear devices. All symbols report the test error at the end of the 50th epoch, and the error bars capture the test error fluctuations for the last five epochs. Lines are guides to the eye. The floating-point baseline is shown with the black horizontal line at 1.32 test error. After random weight initialization, an untrained network gives ∼4.46 test error corresponding to a random guess.

To further examine the resilience of TTv2 to other analog hardware issues, namely the number of states and the cycle-to-cycle update noise, we performed training simulations by varying $\mu_{\Delta w}$ many decades from 0.6 × 10^−4^ to 0.15. This 2,500x increase in $\mu_{\Delta w}$ causes a 2,500x reduction in states’ number on RPU devices from 20,000 down to 8. Furthermore, as $\mu_{\Delta w}$ increases, the amount of noise existing during the pulsed updates increases by 2,500x since cycle-to-cycle noise is defined relative to the state definition on each device as described above. Figure 4 summarizes these simulation results, where the test error at the end of the 50th epoch is reported. For each data point in Figure 4, we finetuned each algorithm’s hyper-parameters independently and reported the best training results. Both SGD and Tiki-Taka are very sensitive to the number of states and the update noise as the test error increases quickly with an increase in $\mu_{\Delta w}$. Whereas the error for TTv2 remains unchanged for many decades and highlights the orders of magnitude increased tolerance of TTv2 to the limited number of states and the update noise. Compared to SGD and Tiki-Taka, TTv2 is at least 100x more resilient to these two common hardware issues that appear during the update cycle on analog arrays.

**FIGURE 5:**
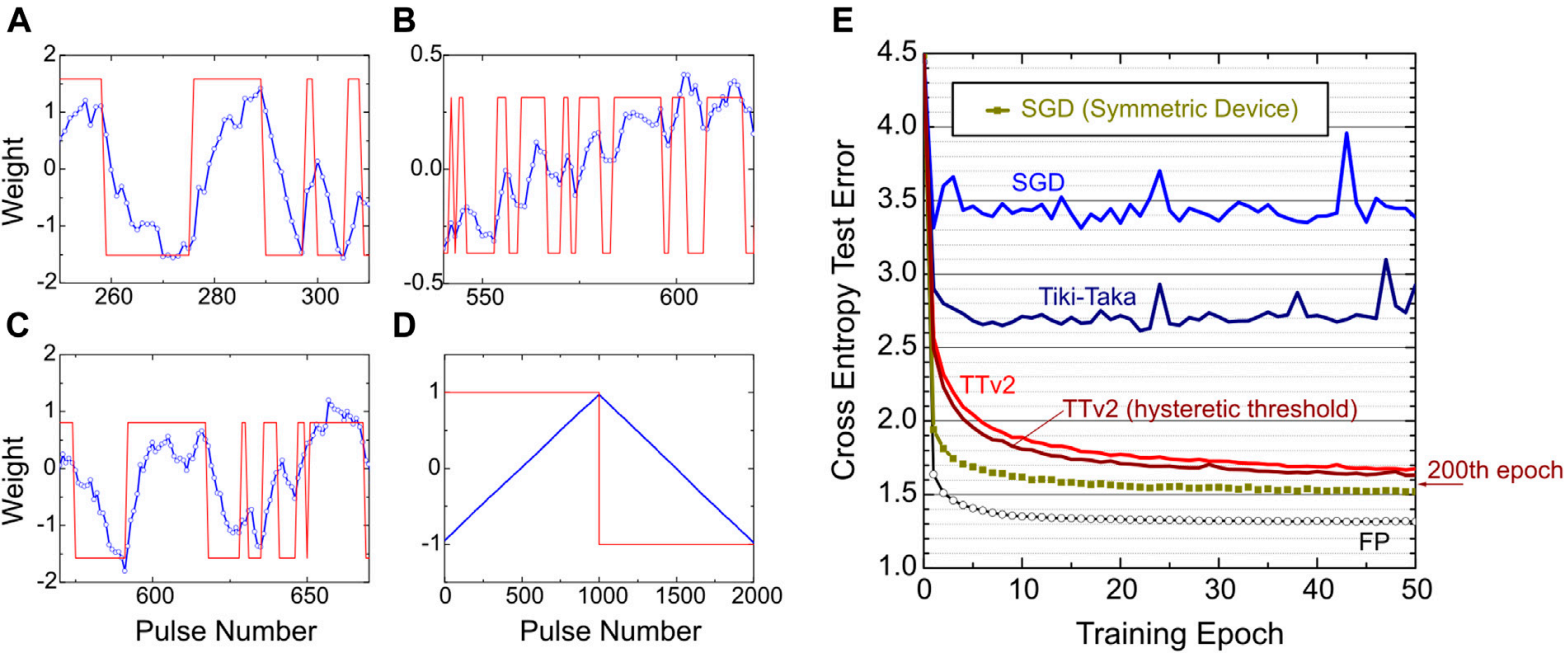
**(A, B, C)** Blue curves: The evolution of three different weights (corresponding to three different devices with non-symmetric behavior, $\sigma_{cycle}=1$ and about 15 states) during TTv2 training. Red curves show the sign of the updates and the expected average saturation value for the corresponding device. **(D)** The evaluation of a linear and symmetric device with $\sigma_{cycle}=0.3$ and more than 1,000 states. **(E)** LSTM training simulations for SGD, Tiki-Taka, and TTv2 algorithms. Different color curves use an extremely noisy array model with non-symmetric devices, $\mu_{\Delta w}=0.08$ (corresponding to 15 states), and the additive cycle-to-cycle update noise with $\sigma_{cycle}=1$. The square symbols show the SGD training baseline from Figure 3 with symmetric device arrays with 1,200 states and cycle-to-cycle noise at $\sigma_{cycle}=0.3$. The open circles are the floating-point baseline.

Finally, in Figure 5, we additionally tested the success of TTv2 at an extremely noisy hardware setting. These simulations assume $\mu_{\Delta w}=0.08$ corresponding to an average of 15 states, but with an even higher cycle-to-cycle update noise setting with the additive noise model at $\sigma_{cycle}=1$. Figures 5A–C illustrate (for three different devices) the amount of update noise and the array level variability used for TTv2. The blue curves show the evolution of the weights after each pulse during training. The red curves show the sign of the updates and the expected average saturation value for the corresponding device for positive and negative pulses. The saturation values are very different due to array level variability, and the response to each pulse is very noisy due to the additive cycle-to-cycle update noise. As a comparison, we also show the response of a linear and symmetric device with $\sigma_{cycle}=0.3$ and more than 1,000 states in Figure 5D. The noise is not even visible for this device used only for the SGD simulations, further emphasizing the burden imposed on the TTv2 algorithm.

The training simulations in Figure 5E show that TTv2 achieves acceptable training results even at these extremely noisy hardware settings. Figure 5E also shows a slightly modified TTv2 implementation with a hysteretic threshold that achieves a better result than TTv2. In this modified TTv2 implementation, we only changed line 20 of TTv2 from $h_{it}=0$ to $h_{it}=sign\left(h_{it}\right)0.6$. This change makes the thresholding event asymmetric and hysteretic: Back to back same sign updates on C happens with a 0.4 threshold, whereas back to back different sign updates must overcome a threshold of 1.6. These hysteretic updates allow the system to correct itself quickly if the previous update caused an undesired modulation on the weight. Note that the update noise is so large that it may even cause a change in the weight opposite to the intended direction, as illustrated in Figures 5A–C. Furthermore, same sign updates are encouraged to accelerate the learning along the dimensions that have higher confidence.

Finally, we emphasize that, in contrast to SGD and Tiki-Taka, TTv2 only fails gracefully at these extremely challenging hardware settings. We note that the continued training further improves the performance of TTv2 until 200 epochs, and a test error of 1.57 is achieved for the modified TTv2. This test error is almost identical to one achieved by the symmetric device SGD baseline with 1,200 states and many orders of magnitude less noise. All these results show that TTv2 is superior to Tiki-Taka and SGD, especially when the analog hardware becomes noisy and provides a very limited number of states on RPU devices.

### Implementation Cost of TTv2

The true benefit of using device arrays for training workloads emerges when the required gradient computation (and processing) step is performed in the array using the RPU device properties. As mentioned in the introduction, the gradient computation is 1/3 of the training operations performed on the weights that the hardware must handle efficiently. Irrespective of the layer type, such as convolution, fully connected, or LSTM, for an *n* × *n* weight matrix in a neural network, each gradient processing step per weight reuse has a computational complexity of *O(n* ^*2*^ *)*. RPU arrays perform the required gradient processing step efficiently at *O(1)* constant time using array parallelism. Specifically, analog arrays deliver *O(1)* time complexity simply because the array has *O(n* ^*2*^ *)* compute resources (RPU devices). In this scheme, each computation is mapped to a resource, and consequently, RPU arrays trade space complexity for time complexity, whereas computational complexity remains unchanged. As a result of this spatial mapping, crossbar-based analog accelerators require a multi-tile architecture design irrespective of the training algorithm so that each neural network layer and the corresponding weights can be allocated on separate tiles. Nevertheless, RPU arrays provide a scalable solution for a spatially mapped weight stationary architecture for training workloads thanks to the nano-scale device concepts.

As highlighted in **Algorithm 2**, TTv2 uses the same tile operations and therefore running TTv2 on array architectures requires no change in the tile design compared to SGD or Tiki-Taka. Assuming the tile design remains unchanged, a pair of device arrays operated differentially with the supporting peripheral circuits, TTv2 (like Tiki-Taka) requires twice more tiles to allocate A and C separately. However, alternatively, the logical A and C values can be realized using only three devices by sharing a common reference, as described in Ref Onen et al. (2021). In that case, logical A and C matrices can be absorbed into a single tile design composed of three device arrays and operated in a time multiplex fashion. This tile design minimizes or even possibly eliminates the area cost of TTv2 and Tiki-Taka compared to SGD.

In contrast to A and C matrices allocated on analog arrays, H does not require any spatial mapping as it is allocated digitally, and it can reside on an off-chip memory. Furthermore, we emphasize that the digital H processing of TTv2 must not be confused with the gradient computation step. For an *n × n* weight matrix in a neural network, the computational complexity of the operations performed on H is only *O(n)*, even for the most aggressive setting of ns = 1. As detailed in **Algorithm 2**, only a single row of H is accessed and processed digitally for ns parallel array update operations on A. Therefore, H processing has reduced computational complexity compared to gradient computation: *O(n)* vs. *O(n* ^*2*^ *)*. This property differentiates TTv2 from other approaches performing the gradient computation in the digital domain with *O(n* ^*2*^ *)* complexity (Nandakumar et al., 2020). Regardless, the digital H processing in TTv2 brings additional digital computation and memory bandwidth requirements compared to SGD or Tiki-Taka. To understand the extra burden introduced by H in TTv2, we must compare it to the burden already handled by the digital components for the SGD algorithm. We argue that the extra burden introduced in TTv2 is usually only on the order of 1/ns, and the digital components required by the SGD algorithm can also handle the H processing of TTv2.

A weight reuse factor (*ws*) for each layer in a neural network is determined by various factors, such as time unrolling steps in an LSTM, reuse of filters for different image portions in a convolution, or simply using mini-batches during training. For an *n × n* weight matrix with a weight reuse factor of *ws*, the compute performed on the analog array is *O(n* ^*2*^ *.ws)*. In contrast, the storage and processing performed digitally for the activations and error backpropagation are usually *O(n.ws)*. We emphasize that these *O(n.ws)* compute and storage requirements are common to TTv2, Tiki-Taka, and SGD and are already addressed by digital components.

The digital filter of TTv2 computes straightforward vector-vector additions and thresholds, which require *O(n)* operations performed only after ns weight reuses. As mentioned above, SGD (likewise Tiki-Taka and TTv2) uses digital units to compute the activations and the error signals, both of which are usually *O(n.ws)*. Therefore, the digital compute needed for the H processing of TTv2 increases the total digital compute by *O(n.ws/ns)*.

Additionally, the filter requires the H matrix to be stored digitally. H is as large as the neural network model and requires off-chip memory storage and access. One may argue that this defeats the purpose of using analog crossbar arrays. However, note that even though the storage requirements for H are *O(n* ^*2*^ *)*, the access to H happens one row at a time, which is *O(n)*. Therefore, as long as the memory bandwidth can sustain access to H, the storage requirement is a secondary concern that can easily be addressed by allocating space on external off-chip memory. This increases the required storage capacity from *O(n.ws)* (only for activations) to *O(n.ws) + O(n* ^*2*^ *)* (activations + H).

Finally, assuming H resides on an off-chip memory, the hardware architecture must provide enough memory bandwidth to access H. As noted in **Algorithm 2**, access to H is very regular, and only a single row of H is needed after ns weight reuses. For SGD (and hence for Tiki-Taka and TTv2), the activations computed in the forward pass are first stored in off-chip memory and then fetched from it to compute the error signals during the backpropagation. The activation store and loads are also usually *O(n.ws)*, and therefore the additional access to H in TTv2 similarly increases required memory bandwidth by about *O(n.ws/ns)*.

In summary, compared to SGD, TTv2 introduces extra digital costs that are only on the order of 1/ns, whereas it brings orders of magnitude relaxation to many stringent analog hardware specs. For instance, ns = 5 provided the best training results for the LSTM network, and for that network, the additional burden introduced to digital compute and memory bandwidth remains less than 20%. For the first convolutional layer of the MNIST problem, ns = 576 is used, making the additional cost negligible (Gokmen and Haensch, 2020). However, we note that the neural networks come in many different flavors, beyond those studied in this manuscript, with different stress points on various hardware architectures. Our complexity arguments should only be used to compare the relative overhead of TTv2 compared to SGD, assuming a fixed analog crossbar-based architecture and particular neural network layers. Detailed power/performance analysis of TTv2 with optimized architecture for a broad class of neural network models requires additional studies.

### PART II: Model Extraction

Machine learning experts try various neural network architectures and hyper-parameter settings to obtain the best performing model during model development. Therefore, accelerating the DNN training process is extremely important. However, once the desired model is obtained, it is equally important to deploy the model in the field successfully. Even though training may use one set of hardware, numerous users likely run the deployed model on several hardware architectures, separate from the one the machine learning experts trained the model with. Therefore, to close the development and deployment lifecycle, the desired model must be extracted from the analog hardware for its deployment on another hardware.

In contrast to digital solutions, the weights of the model are not directly accessible on analog hardware. Analog arrays encode the model’s weights, and the tile’s noisy mat-vec limits access to these weight matrices. Therefore, the extraction of the model from analog hardware is a non-trivial task. Furthermore, the model extraction must produce a good representation of the trained model to be deployed without loss of accuracy on another analog or a completely different digital hardware for inference workloads.

In Part II, we first provide how an accurate weight extraction can be performed from noisy analog hardware. Then we further generalize this method to obtain an accurate model average over the TTv2 iterates. Ref Izmailov et al. (2019a) showed that the Stochastic Weight Averaging (SWA) procedure that performs a simple averaging of multiple points along the trajectory of SGD leads to better generalization than conventional training. Our analog-hardware-friendly SWA on TTv2 iterates shows that these techniques inspired by the Bayesian treatment of neural networks can also be applied to analog training hardware successfully. We show that the model averaging further boosts the extracted model’s generalization performance and provides a model that is even better than the trained model itself, enabling the deployment of the extracted model virtually on any other hardware.

### Accurate Weight Extraction

Analog tiles perform mat-vec on the stored matrices. Therefore, naively one can perform a series of mat-vecs using one-hot encoded inputs to extract the stored values one column (or one row) at a time. However, this scheme results in a very crude estimation of the weights due to the mat-vec noise and limited ADC resolution. Instead, we perform a series of mat-vecs using random inputs and then use the conventional linear regression formula, Eq. 4, to estimate the weights.

$$\hat{C}={\left({\left(XX^{T}\right)}^{-1}XY^{T}\right)}^{T}$$ (4)

In Eq. 4, $\hat{C}$ is an estimate of the ground truth matrix C stored on the tile, X has the inputs used during weight extraction, and Y has the resulting outputs read from the tile. Both X and Y are written in matrix form, capturing all the mat-vecs performed on the tile.

**FIGURE 6:**
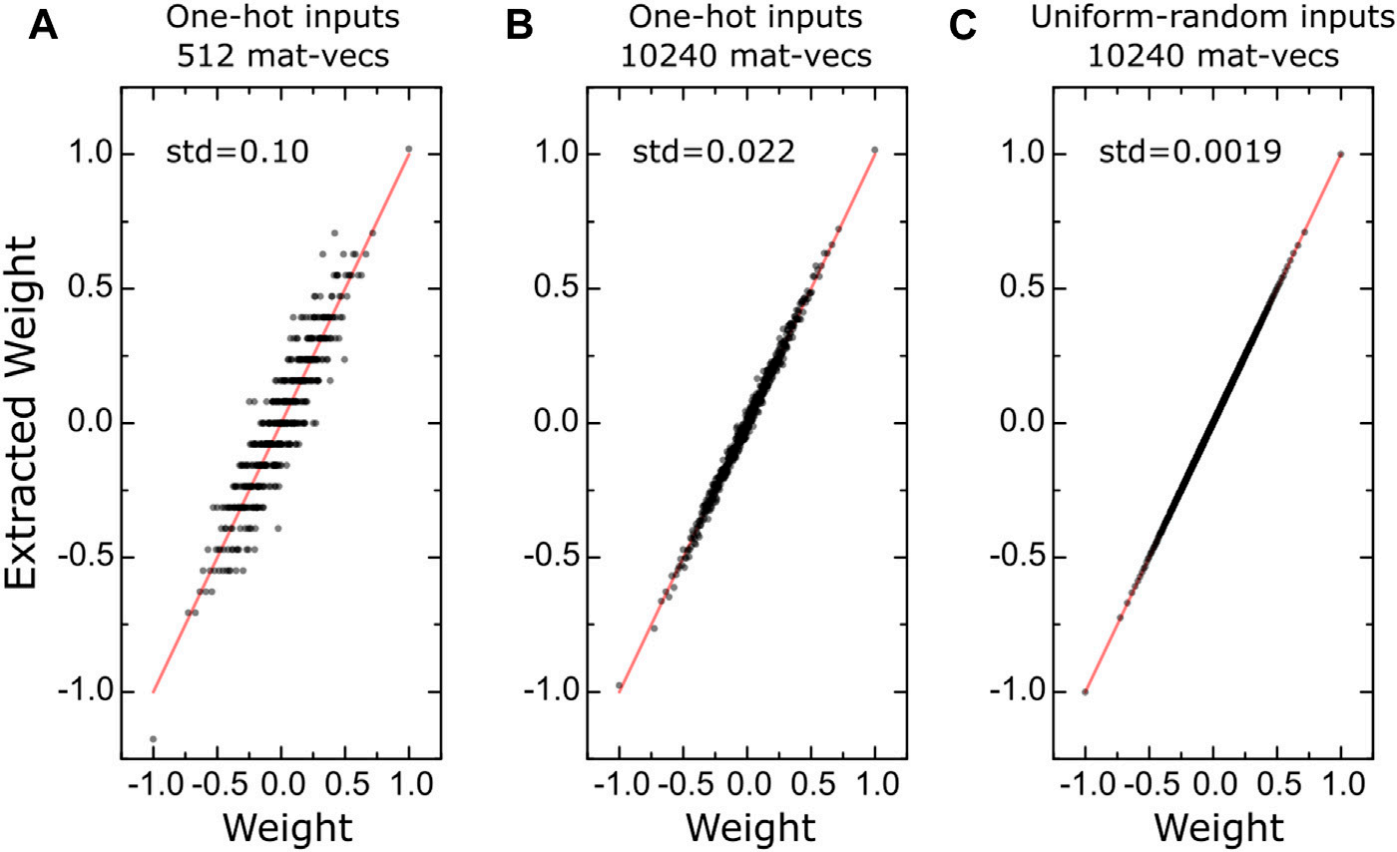
(**A–C**) Correlation between the ground truth weights and the extracted values using different input forms and number of mat-vecs for a simulated 512 × 512 tile. Red lines are guides to the eye showing perfect correlation.

Figure 6 shows the quality of different weight estimations for a simulated tile of size 512 × 512 with the same analog array assumptions described in Part I. When one-hot encoded input vectors are used only once, corresponding to 512 mat-vecs, the correlation of the extracted values to the ground truth is very poor due to analog mat-vec noise ($\sigma_{MV})$ and ADC quantization, as seen in Figure 6A. Repeating the same measurements 20 times, corresponding to a total of 10,240 mat-vecs, improves the quality of the estimate (Figure 6B). However, the best estimate is obtained when completely random inputs with uniform distribution are used, as illustrated in Figure 6C. We note that the total number of mat-vecs is the same for Figures 6B,C, and yet Figure 6C provides a much better estimate. This is because the completely random inputs have the highest entropy (information content), and therefore they provide the best estimate of the ground truth for the same number of mat-vecs.

Note, in this linear regression formalism, the tile noise and quantization error correspond to aleatoric uncertainty and cannot be improved. However, the weight estimates are not limited by the aleatoric uncertainty; and instead, the epistemic uncertainty limits these estimates. For the data shown in Figure 6C, the standard deviation in weight estimation (corresponding to the epistemic uncertainty) is 0.002, only 0.1% of the nominal weight range of [−1, 1] used for these experiments. The uncertainty in weight estimates scales with $1/\sqrt{\text{number}\_\text{of}\_\text{mat}\_\text{vecs}}$, and if needed, this uncertainty can be further reduced by performing more measurements.

### Accurate Model Average

As shown in Ref Izmailov et al. (2019a), SWA performs a simple averaging of multiple points along the trajectory of SGD and leads to better generalization than conventional training. This SWA procedure approximates the Fast Geometric Ensemble (FGE) approach with a single model. Furthermore, Ref Yang et al. (2019) showed that SWA brings benefits to low precision training. Here, we propose that weight averaging over TTv2 iterates would also bring similar gains and possibly overcome noisy updates unique to the RPU devices. However, obtaining the weight averages from analog hardware may become prohibitively expensive. Naïvely, the weights can be first extracted from analog hardware after each iteration and then accumulated in the digital domain to compute averages. However, this requires thousands of mat-vecs per iteration and therefore is not feasible.

Instead, to estimate the weight averages, we perform a series of mat-vecs that are very sparse in time but performed while the training progresses and then use the same linear regression formula to extract the weights. Since the mat-vecs are performed while weights are still evolving, the extracted values closely approximate the weight averages for that training period. For instance, during the last 10 epochs of the TTv2 iterates, we performed 100 K mat-vecs with uniform-random inputs and showed that it is sufficient to estimate the actual weight averages with less than 0.1% uncertainty.

We note that about 60 M mat-vecs on C and 30 M updates on A are performed during 10 epochs of training. Therefore, the additional 100 K mat-vecs on C needed for weight averaging increases the compute on the analog tiles by only 0.1%. Furthermore, the input and output vectors (x,y) for each mat-vec can be processed on the fly by accumulating the results of $xx^{T}$ and $xy^{T}$ on two separate matrices in the digital domain: $M_{xx}\leftarrow M_{xx}+xx^{T}$ and $M_{xy}\leftarrow M_{xy}+xy^{T}$. Then at the end of the training, one matrix inversion and a final matrix-matrix multiply need to be performed to complete all the steps needed to estimate the weight averages: ${\hat{C}}_{avg}={\left({\left(M_{xx}\right)}^{-1}M_{xy}\right)}^{T}$.

In practical applications, a separate conventional digital processor (like CPU) can perform the computations needed for weight averages by only receiving the results of the mat-vecs from the analog accelerator. Note that the CPU can generate the same input vectors by using the same random seed. Therefore, $M_{xx}$ and its inverse can be computed and stored well ahead of time, even before training starts. Furthermore, the same input vectors and a common ${\left(M_{xx}\right)}^{-1}$ can extract the weight averages from multiple analog tiles. After all these optimizations, even a conventional digital processor can sustain the computation needed for $M_{xy}$ from multiple tiles and provide the weight averages at the end of training.

### Inference Results

To test the validity of the proposed weight extraction and averaging techniques, we study the same model trained on extremely noisy analog hardware using TTv2 with the hysteretic threshold. We refer to this model as Model-I. As shown in Figure 5E, the test error of Model-I at the end of the 50th and 200th epochs are 1.633 and 1.570, respectively. These test errors assume Model-I runs inferences on the same analog hardware it trained on and form our baseline.

We apply our model extraction technique in the first experiment and obtain the weights using only 10 K mat-vecs with random inputs. We refer to this extracted model as Model-I_x_, and it is an estimate of Model-I. We evaluate the test error of Model-I_x_ when it runs either on another analog hardware (with the same analog array properties) or digital hardware. As summarized in Table 1, Model-I_x_’s test error remains unchanged on the new analog hardware compared to Model-I, showing our model extraction technique’s success. Interestingly, the inference results of Model-I_x_ are better on the digital hardware, and the test errors drop to 1.583 and 1.524 respective for the 50th and 200th epochs. These improvements are due to the absence of the mat-vec noise introduced by the forward propagation on analog hardware. However, these results also highlight that the analog training yields a better model than the test error on the same analog hardware indicates. Therefore, such benefits ease analog hardware’s adoption for training only purposes, and the improved test results on digital hardware are the relevant metrics for such a use case.

**TABLE 1:** Inference results of various models on different hardware.

	Model–I, I_x_, I_avg_#States = 15, $\sigma_{cycle}=1$	Model–II, II_x_, II_avg_#States = 60, $\sigma_{cycle}=0.3$	Model–III,III_x_, III_avg_#States = 120, $\sigma_{cycle}=0.3$
Inference on Analog Hardware			
50th Epoch-Trained Model	**1.633**	**1.454**	**1.430**
50th Epoch-Extracted Model	1.633	1.455	1.430
40th–50th Epochs-Extracted Model Avg	1.560	1.425	1.407
200th Epoch-Trained Model	**1.570**	**1.410**	**1.403**
200th Epoch-Extracted Model	1.571	1.410	1.403
180th–200th Epochs-Extracted Model Avg	1.487	1.377	1.372
Inference on Digital Hardware			
50th Epoch-Extracted Model	1.583	1.403	1.378
40th–50th Epochs-Extracted Model Avg	1.520	1.379	1.359
200th Epoch-Extracted Model	1.524	1.360	1.350
180th–200th Epochs-Extracted Model Avg	1.454	*1.334*	*1.326*

FP Baseline Model: 1.315–1.332.

Repeating the same FP training results in about 0.01 variability in the test error due to the randomness in weight initialization. Bold values provide the baseline training results without model extraction. Italic values correspond to models that are indistinguishable from the FP model.

We implement our model averaging technique using 100 K mat-vecs with random inputs applied between 40–50 or 180–200 epochs in the following experiment. We refer to the extracted model average as Model-I_avg_, and the test error for Model-I_avg_ is also evaluated on analog or digital hardware. In all cases, as illustrated in Table 1, Model-I_avg_ gives non-trivial improvements compared to Model-I_x_ (and Model-I). These improvements on the averaged models’ generalization performance show the success of our model averaging technique. We emphasize that the model training is performed on extremely noisy analog hardware using TTv2. Nevertheless, the test error achieved by Model-I_avg_ on digital hardware is 1.454, just shy of the FP model’s performance at about 1.325.

Finally, to further illustrate the success of the proposed model extraction and averaging techniques, we performed simulations for another two models, Model II and III, which are also summarized in Table 1. Like Model-I, these models are also trained on noisy analog hardware but with slightly relaxed array assumptions. The only two differences compared to Model-I are 1) Model-II and III both used analog arrays with the additive cycle-to-cycle update noise at $\sigma_{cycle}=0.3$, 2) Model-II and III respectively had 60 and 120 states on RPU devices. For these slightly relaxed but still significantly noisy analog hardware settings, both Model-II and III provide test results on the digital hardware that are virtually indistinguishable from the FP model when the model averages between 180–200 epochs are used.

We note that the inference simulations performed on analog hardware did not include any weight programming errors that may otherwise exist in real hardware. Depending on its strength, these weight programming errors cause an accuracy drop on the analog hardware used solely for inference purposes. Additionally, after the initial programming, the accuracy may further decline over time due to device instability, such as the conductance drift (Mackin et al., 2020; Joshi et al., 2020). Therefore, any analog hardware targeting inference workloads must address these non-idealities. However, we emphasize that these problems are unique to inference workloads. Instead, if analog hardware is targeting training workloads only, these problems become obsolete. Furthermore, the unique challenges of the analog training hardware, namely the limited number of states on RPU devices and the update noise, are successfully handled by our proposed TTv2 training algorithm and the model averaging technique. As illustrated above, even very noisy analog hardware can deliver models on par in their accuracy compared to FP models. In addition, after the training process is performed on analog hardware using TTv2, the extracted model average can be deployed on various digital hardware and perform inference without any accuracy loss. Therefore, these results provide a clear path for analog hardware to be employed to accelerate DNN training workloads.

## Discussion and Future Directions

DNN training using SGD is simply an optimization algorithm that provides a point estimate of the DNN parameters at the end of the training. In this frequentist view, a hypothesis is tested without assigning any probability distribution to the DNN parameters and lacks the representation of uncertainty. More recently, however, the Bayesian treatment of DNNs has gained more traction with new approximate Bayesian approaches (Wilson, 2020). Bayesian approaches treat the DNN parameters as random variables with probabilities. We believe many exciting directions for future research may connect these approximate Bayesian approaches and neural networks running on noisy analog hardware.

For instance, Ref Maddox et al. (2019) showed that a simple baseline for Bayesian uncertainty could be formed by determining the weight uncertainties from the SGD iterates, referred to as SWA-Gaussian. It is empirically shown that SWA-Gaussian approximates the shape of the true posterior distribution of the weights, described by the stationary distribution of SGD iterates. We can intuitively generalize these results to the TTv2 algorithm running on analog hardware. For instance, the proposed TTv2 algorithm updates a tiny fraction of the neural network weights when enough evidence is accumulated by A and H’s gradient processing steps. Nevertheless, the updates on weights are still noisy due to stochasticity in analog hardware. Therefore, TTv2 iterates resemble the Gibbs sampling algorithm used to approximate a posterior multivariate probability distribution governed by the loss surface of the DNN. Assuming this intuition is correct, analyzing the uncertainty in weights over TTv2 iterates may provide a simple Bayesian treatment of a DNN, similar to SWA-Gaussian.

To test the feasibility of the above arguments, we performed the following experiments that are motivated by the results of SWA-Gaussian (Maddox et al., 2019) and Bayes-by-Backprop (Blundell et al., 2015): First, we extract the mean ($\mu_{i}$) and the standard deviation ($\sigma_{i}$) of each weight from the TTv2 iterates and define a signal-to-noise ratio as $\left|\mu_{i}\right|/\sigma_{i}$. Then we remove the weights with the lowest signal-to-noise ratio below a certain value and compare the inference performance of this *carefully* pruned network to the unpruned one. We also look at the performance degradation of a randomly pruned network with the same amount of weight pruning. Table 2 summarizes the results of these experiments performed for Model-III from 180 to 200 epochs.

**TABLE 2:** Inference results of pruned networks for Model-III on digital hardware.

Signal-to-noise used for pruning	Weight proportion removed (%)	Carefully pruned network	Randomly pruned network
(No pruning)	0	1.326	1.326
$\left|\mu_{i}\right|/\sigma_{i}<1$	16.7	1.331	3.42 ± (0.26)
$\left|\mu_{i}\right|/\sigma_{i}<2$	30.2	1.371	4.09 ± (0.32)
$\left|\mu_{i}\right|/\sigma_{i}<3$	40.8	1.466	4.40 ± (0.29)

Random pruning experiments are performed 10 times. The table reports the mean and standard deviation of these 10 experiments for the randomly pruned networks. An untrained network gives ∼4.46 test error corresponding to a random guess.

As illustrated in Table 2, the *carefully* pruned network’s performance (1.331) is almost identical to the unpruned one (1.326) when $\left|\mu_{i}\right|/\sigma_{i}<1$, corresponding to 16.7% pruning. However, the same amount of pruning causes significant performance degradation for a randomly pruned network (∼3.42). When the signal-to-noise threshold is raised to 3, corresponding to 40.8% pruning, the *carefully* pruned network still performs reasonably well (1.466). Whereas at this level of pruning, a randomly pruned network is not any better than an untrained network producing random predictions.

In the second set of experiments, as summarized in Table 3, we use the extracted means ($\mu_{i}$) and standard deviations ($\sigma_{i}$) and disturb each weight randomly proportional to its standard deviation: $w_{i}=\mu_{i}+\xi \sigma_{i}$, where ξ is sampled from a unit Gaussian for each weight. Then, we compare the inference performance of this *carefully* disturbed network to a randomly disturbed network with the same amount of total weight disturbance. Although the *carefully* disturbed network performs reasonably well at 1.493, the randomly disturbed networks’ performance significantly degrades to about 3.54.

**TABLE 3:** Inference results of disturbed networks for Model-III on analog hardware.

Signal-to-noise used for disturbance	Carefully disturbed network	Randomly disturbed network
(No disturbance)	1.370	1.370
$\mu_{i}\pm \sigma_{i}$	1.493	3.54 ± (0.15)

Random disturb experiments are performed 10 times. The table reports the mean and standard deviation of these 10 experiments.

These experiments empirically suggest that the weight uncertainty of TTv2 iterates on analog hardware provides additional valuable information about the posterior probability distribution of the weights governed by the loss surface of the DNN. The results illustrated in Tables 2, 3 do not address how the weight uncertainty can be extracted from analog hardware in practical settings; however, suppose this information can be extracted. In that case, the weight uncertainty can be used to sparsify the DNN during the model deployment on digital hardware (Blundell et al., 2015). Alternatively, the weight uncertainties can be leveraged to devise better programming routines while transferring the model to another noisy analog hardware. In addition, a low dimensional subspace can be constructed over TTv2 iterates so that the model can be deployed as a Bayesian neural network, similar to the results presented in Ref Izmailov et al. (2019b). The Bayesian model averaging performed even in low dimensional subspaces produces accurate predictions and well-calibrated predictive uncertainty (Izmailov et al., 2019b). We believe that noisy analog hardware with modified learning algorithms can also accelerate Bayesian approaches while simultaneously providing many known benefits, such as improved generalization and uncertainty calibration. However, these ideas require further investigation, and new techniques that can also extract the weight uncertainty from analog hardware are needed. Furthermore, extending this work to larger and more extensive networks is a general task for the feasibility of analog crossbar arrays, not only restricted to the work presented here.

## Summary

In summary, we presented a new DNN training algorithm, TTv2, that provides successful training on extremely noise analog hardware composed of resistive crossbar arrays. Compared to previous solutions, TTv2 addresses all sorts of hardware non-idealities coming from resistive devices and peripheral circuits and provides orders of magnitude relaxation to many hardware specs. Device arrays with non-symmetric and noisy conductance modulation characteristics and a limited number of states are enough for TTv2 to train neural networks close to their ideal accuracy. In addition, the model averaging technique applied over TTv2 iterates provides further enhancements during the model extraction. In short, we describe an end-to-end training algorithm and model extraction technique from extremely noisy crossbar-based analog hardware that matches the performance of full-precision SGD training. Our techniques can be immediately realized and applied to many readily available device technologies that can be utilized for analog deep learning accelerators.

## Data Availability

The original contributions presented in the study are included in the article/supplementary material, further inquiries can be directed to the corresponding author.
